# Altered huntingtin−chromatin interactions predict transcriptional and epigenetic changes in Huntington's disease

**DOI:** 10.1242/dmm.052282

**Published:** 2025-05-27

**Authors:** Jocelynn R. Pearl, Amol C. Shetty, Jeffrey P. Cantle, Dani E. Bergey, Robert M. Bragg, Sydney R. Coffey, Holly B. Kordasiewicz, Leroy E. Hood, Nathan D. Price, Seth A. Ament, Jeffrey B. Carroll

**Affiliations:** ^1^ Institute for Systems Biology, Seattle, WA 98109, USA; ^2^ Molecular & Cellular Biology Graduate Program, University of Washington, Seattle, WA 98195, USA; ^3^ Institute for Genome Sciences, University of Maryland School of Medicine, Baltimore, MD 21201, USA; ^4^ Behavioral Neuroscience Program, Department of Psychology, Western Washington University, Bellingham, WA 98225, USA; ^5^ Department of Neurology, University of Washington, Seattle, WA 98195, USA; ^6^ Ionis Pharmaceuticals, Carlsbad, CA 92010, USA; ^7^ Buck Institute for Research on Aging, Novato, CA 94945, USA; ^8^ Phenome Health, Seattle, WA 98109, USA; ^9^ Thorne HealthTech, New York, NY 10019, USA; ^10^ Maryland Psychiatric Research Center, Department of Psychiatry, University of Maryland School of Medicine, Baltimore, MD 21201, USA

**Keywords:** Huntington's disease, Huntingtin, Chromatin, Epigenetics

## Abstract

While progressive striatal gene expression changes and epigenetic alterations are a prominent feature of Huntington's disease (HD), the mechanistic basis remains poorly understood. Using chromatin immunoprecipitation and sequencing (ChIP-seq), we show that the huntingtin protein (HTT) reproducibly occupies specific locations in the mouse genome. Striatal HTT ChIP-seq peaks were enriched in coding regions of spiny projection neuron identity genes that were found to have reduced expression in HD patients and mouse models, and had reduced occupancy in expanded polyglutamine HTT knock-in mice (*Htt^Q111/Q111^*). By contrast, HTT occupancy was depleted near genes that are upregulated in HD. ChIP-seq of striatal histone modifications revealed genotype-specific colocalization of HTT with active chromatin marks and enhancer of zeste homolog 2 (EZH2), a key enzymatic component of the PRC2 complex. In the vicinity of genes that are differentially regulated in HD, greater HTT occupancy in *Htt^Q111/Q111^* vs wild-type mice was associated with increased EZH2 occupancy, increased H3K4me3 levels and decreased H3K27me3 levels. Our study suggests that HTT−chromatin interactions may play a role in organizing chromatin and promoting cell type-specific gene expression, with HTT occupancy predicting transcriptional dysregulation in HD.

## Teaser

**Summary:** Huntingtin chromatin occupancy supports a model in which perturbation of its normal functions leads to epigenetic changes and precipitates the loss of cell type-specific gene expression seen in Huntington's disease mouse models.

## INTRODUCTION

Huntington's disease (HD) is a fatal dominant neurodegenerative disease caused by expansion of a glutamine-coding (polyQ) CAG tract near the 5′ end of the *Huntingtin* (*HTT*) gene (The Huntington's Disease Collaborative Research Group, 1993). Clinical symptoms include deficits in motor control and cognition, as well as psychiatric symptoms. HD progression is linked to the selective death of spiny projection neurons (SPNs) in the striatum (Bates et al., 2015). Transcriptional (Cha, 2007) and epigenomic (Achour et al., 2015; Bassi et al., 2017; Le Gras et al., 2017; Alcalá-Vida et al., 2021) dysregulation are among the earliest phenotypes in cells and tissues expressing mutant HTT protein (mHTT), an observation that is reproducible and shared between brain tissue from humans with HD and in mouse models (Ament et al., 2017; Hodges et al., 2006; Langfelder et al., 2016). While the molecular mechanisms by which mHTT mediates these changes are incompletely understood, the importance of somatic CAG expansion (De Rooij et al., 1995; Kono et al., 1999; Telenius et al., 1994; Mätlik et al., 2024) passing a critical threshold is now beginning to be appreciated as a major pathogenic milestone (Handsaker et al., 2025; Wang et al., 2025).

A straightforward hypothesis is that HTT directly contributes to transcriptional dysregulation in HD through interactions with chromatin-bound DNA. In the cell, HTT protein dynamically shuttles between the cytoplasm and nucleus (Xia et al., 2003). Mis-localization of mHTT to the nucleus is an early phenotype in HD animal models, roughly coincident with the onset of transcriptional changes, and a critical driver of mHTT-mediated neuronal death, both *in vitro* and in mouse models (Xia et al., 2003; Gu et al., 2015; Saudou et al., 1998). To date, specific interactions of HTT with chromatin remain obscure but are supported by several lines of indirect evidence: (i) chromatin immunoprecipitation indicates that HTT affiliates with chromatin DNA (Benn et al., 2008); (ii) the HTT protein contains a series of HEAT domains, which are capable of serving as DNA-binding domains (Neuwald and Hirano, 2000); and (iii) HTT forms direct protein−protein interactions with a variety of transcriptional regulatory proteins in the nucleus, including transcription factors and chromatin remodeling factors (Saudou and Humbert, 2016). Perhaps the best understood of such interactions involve chromatin remodeling complexes that mediate gene repression and heterochromatin formation. HTT binds in a polyQ-length-sensitive manner to polycomb repressive complex 2 (PRC2), the chromatin remodeling complex responsible for trimethylation of lysine 27 on the histone 3 tail (H3K27me3), a repressive mark associated with bivalent or poised chromatin regions that are crucial for developmental fate commitment (Seong et al., 2010). Ablation of PRC2 in SPNs reproduces several of the cellular phenotypes in HD, including aberrant de-repression of transcripts that encode developmentally regulated transcription factors, loss of SPN identity gene expression and prolonged cell death (von Schimmelmann et al., 2016). We (Malaiya et al., 2021) and others (Achour et al., 2015; Langfelder et al., 2016) have previously observed that a common transcriptional feature of HD is a reduction of cell-type-appropriate gene expression, providing additional impetus to understand changes in PRC2 function in HD, given its key role in the regulation of cell fate (Margueron and Reinberg, 2011).

To investigate the hypothesis that HTT occupies specific locations on chromatin DNA, we performed chromatin immunoprecipitation and deep sequencing (ChIP-seq) to map HTT genomic occupancy in striatal tissue obtained from the *Htt^Q111/Q111^* knock-in mouse model of the HD mutation and in wild-type *Htt^+/+^* controls. We analyzed these data together with publicly available RNA-seq and newly generated ChIP-seq data of the histone modifications H3K27me3, H3K9me3 and H3K4me3, and the PRC2 histone methyltransferase effector subunit EZH2, generated in parallel from the striatum of age-matched of *Htt^Q111/+^* mice. We describe thousands of reproducible HTT ChIP-seq peaks, both in *Htt^+/+^* and *Htt^Q111/Q111^* mice, and observe robust genotype-specific patterns of occupancy that are correlated with epigenetic and transcriptional changes seen in HD. These results provide, for the first time, a genome-wide map of HTT genomic occupancy and suggest that altered transcription in HD arises, in part, via alteration of direct HTT−chromatin interactions.

## RESULTS

### HTT reproducibly occupies thousands of locations in the mouse genome

**Fig. 1.**
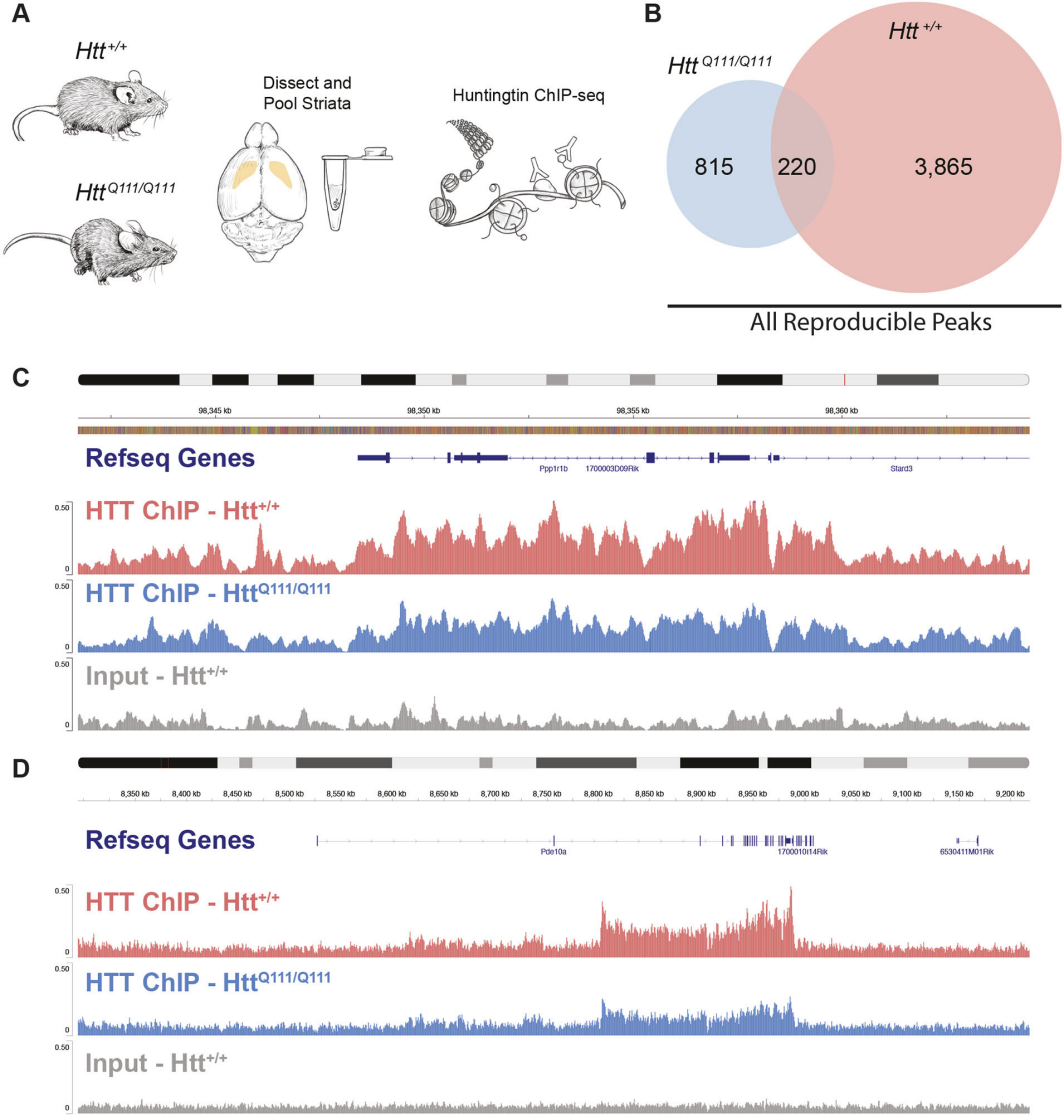
**Reproducible HTT ChIP-seq peak profiles of 4-month-old *Htt^+/+^* and *Htt^Q111/Q111^* mice.** (A) Schematic of experimental layout. (B) Venn diagram depicting the number *Htt^+/+^*-specific, *Htt^Q111/Q111^*-specific and shared HTT peaks reproducible at FDR<0.01 in at least two biological replicates from each genotype. (C,D) Normalized genomic coverage at representative HTT ChIP-Seq peaks in *Htt^+/+^* mice (peach) and *Htt^Q111/Q111^* mice (blue), with *Htt^+/+^* input control sample (gray). Chromosomal locations and NCBI Reference Sequence Database (Refseq Genes) (mouse genome mm10) are indicated. Genes upstream of *Pde10a* (D) have been omitted for labeling clarity.

We set out to map the genomic occupancy of HTT and assess its relationship to HD mutations in the striatum of *Htt^Q111/Q111^* and wild-type mice. *Htt^Q111^* is a well-characterized, genetically precise knock-in mouse model of a mutation associated with juvenile-onset HD, in which a human allele of *HTT* exon 1 with approximately 111 glutamine-encoding CAG repeats has been inserted into the endogenous mouse *Htt* locus. For maximal fidelity to genetics of human HD patients, most studies utilize heterozygous *Htt^Q111/+^* mice. However, for HTT ChIP-seq experiments homozygous *Htt^Q111/Q111^* mice are preferred to avoid the confound of two isoforms of HTT being present in each sample*.* HD is inherited in a fully dominant fashion, and HD patients with pathogenic-length CAG repeat expansions in two alleles generally experience symptoms equivalent to heterozygous patients with a single mutant allele. Likewise, the progression of disease-related phenotypes is comparable in heterozygous *Htt^Q111/+^* mice and *Htt^Q111/Q111^* homozygotes, with four months of age representing an early time point. At this age, we (Ament et al., 2017; Bragg et al., 2017) and others (Langfelder et al., 2016) have detected hundreds of differentially expressed genes in striatal tissue and misfolded HTT isoforms in the nuclei of many striatal SPNs from *Htt^Q111/+^* mice, but there is not yet any discernible striatal cell death or glial proliferation (Bragg et al., 2017). Chromatin immunoprecipitation and deep sequencing (ChIP-seq) has been performed using striatal tissue from 4-month-old *Htt^Q111/Q111^* and wild-type mice, using the well-validated antibody EPR5526 (Schut et al., 2015) that recognizes an N-terminal epitope of the HTT protein with no known differences in affinity for wild-type vs mutant HTT isoforms. We sequenced three biological replicates from *Htt^Q111/Q111^* mice and three from *Htt^+/+^* mice, with each biological replicate consisting of pooled striata from three mice (Fig. 1A).

Initial inspection suggested that HTT occupies broad domains along chromatin DNA (e.g.Fig. 1C-D). We conducted peak-calling using model-based analysis of ChIP-seq data (MACS) (Zhang et al., 2008) in broad peak mode, which revealed 9656 reproducible peaks, of which 9624 peaks were not overlapping blacklisted regions and 4900 peaks were conditionally reproducible, i.e. identified independently in at least two biological replicates from each genotype (Fig. 1B; Tables S1, S2). Reproducible HTT peaks (HTT, mHTT and shared) were found in both genic and intergenic regions: 0.1% in the 5′ UTR; 31% within 3 kb upstream of the transcriptional start site (TSS); 1.1% in the first exon; 3.6% in other exons; 10.7% in the first intron; 28.1% in other introns; and 4.4% in the 3′ UTR, with 20.8% ≤300 kb downstream or in distal intergenic regions (Fig. 4A). A HTT-sequence-specific control experiment supports the hypothesis that these regions accurately reflect HTT occupancy rather than non-specific signals. Namely, we observed fewer HTT ChIP-seq peaks in brain tissue from mice treated with an antisense oligonucleotide (ASO) to reduce HTT levels in the brain (Fig. S1).

Robust HTT peaks were found within 1 Mb of 2628 protein-coding genes. Importantly, most genes do not contain HTT-binding sites, suggesting that HTT occupancy in the vicinity of a given gene may reflect a specific regulatory role limited to this subset of genes, rather than promiscuous binding to every gene. Manual examination of the HTT peaks in gene bodies shows greater HTT occupancy near the 3′ end of the coding region, rather than binding as a sharp peak in a promoter or enhancer, as would be expected if HTT acts as a transcription factor (Benn et al., 2008). Genes with this HTT occupancy pattern include canonical markers for SPNs, such as *Ppp1r1b* (DARPP32) and *Pde10a* (Fig. 1C-D), which have previously been shown to have altered chromatin conformation in the striatum of *Htt^Q140/Q140^*mice (Alcalá-Vida et al., 2021).

### Differential occupancy in *Htt^Q111/Q111^* mice

**Fig. 2.**
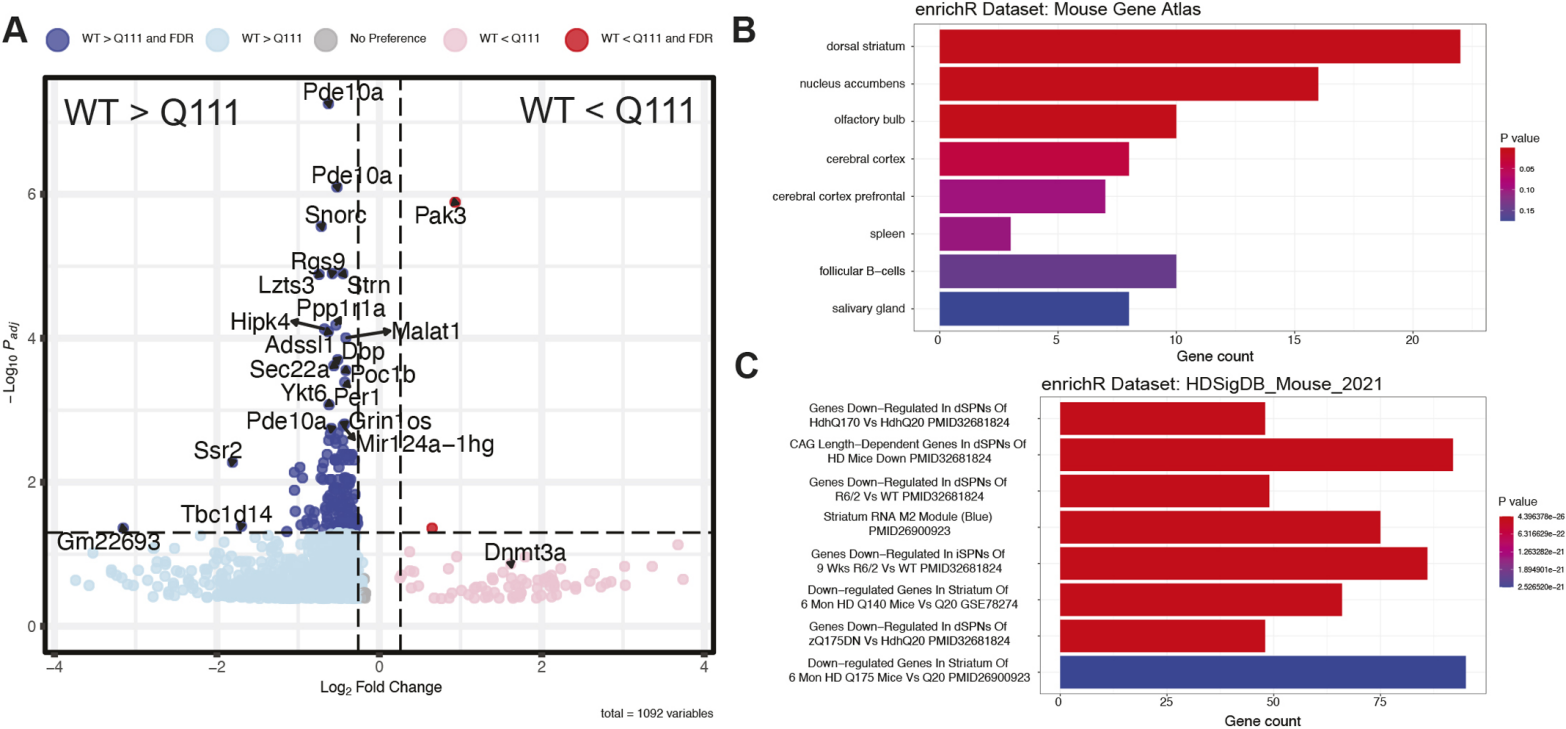
**Differential chromatin binding of HTT in *Htt^+/+^* and *Htt^Q111/Q111^* mice.** (A) Volcano plot summarizing the results of the DiffBind analysis. The *x*-axis indicates the fold change, the *y*-axis the adjusted *P*-value. DiffBind peaks with an FDR<0.05 and/or an absolute fold change of >0.26 are identified by the nearest gene symbol. DiffBind peaks not matching those criteria are indicated by gray dots. (B) 244 genes with reduced peaks in the *Htt^Q111/Q111^* mice (FDR<0.1) were examined for enrichment amongst a number of different mouse tissues by using Enrichr. The bar graph indicates the count of these genes amongst mouse tissues with significant enrichments. Only the terms ‘dorsal striatum’ and ‘nucleus accumbens’ meet the stringency threshold *P_adj_*<0.05. (C) Enrichment of DiffBind nominated peaks in HDSigDB gene sets reveals strikingly enriched sets, notably those that include genes that are downregulated in the striatum of HD mouse models.

Next, we quantified HTT occupancy in *Htt^Q111/Q111^* vs wild-type mice using DiffBind (Ross-Innes et al., 2012). We found 244 peaks with significantly reduced occupancy in *Htt^Q111/Q111^* compared to wild-type mice and four peaks with higher occupancy in *Htt^Q111/Q111^* mice [False Discovery Rate (FDR)<0.1 (Fig. 2A; Table S3), suggesting a bias towards reduced occupancy in *Htt^Q111/Q111^* samples. Notably, genes with nearby reduced peak occupancy in *Htt^Q111/Q111^* striata include important SPN identity genes − many with robust HTT ChIP-seq signals across the coding region − such as *Pde10a* and *Rgs9* (Fig. 2A). To more formally establish the gene sets associated with genes near to DiffBind-nominated peaks, we assigned peaks to the nearest annotated gene if they were overlapping with the TSS (−1 kb to +100 bp), TTS (−100 bp to +1 kb), exon, intronic or intergenic region (1 kb to 1Mb), and conducted enrichment analyses using Enrichr (Xie et al., 2021). Genes near HTT peaks that are reduced in *Htt^Q111/Q111^* mice relative to *Htt^+/+^* mice were more likely to be markers of striatal identity, as cataloged by the Mouse Gene Atlas (Wu et al., 2016) (Fig. 2B; Table S4). No such enrichments were found for genes near HTT peaks that are higher in *Htt^Q111/Q111^* relative to *Htt^+/+^* mice (data not shown). Next, we investigated enrichments in a collection of 2579 manually curated HD-relevant transcriptional signatures from the Huntington's Disease Molecular Signatures Database (HDSigDB; https://hdsigdb.hdinhd.org/, 2021 mouse version). Genes near HTT peaks with decreased occupancy in *Htt^Q111/Q111^* mice were markedly enriched, with genes found to be downregulated in the striatum of preclinical HD mouse models (e.g. ‘Genes Down-Regulated In dSPNs Of HdhQ170 Vs HdhQ20 PMID32681824’; *p_adj_*=8.45e-23; Fig. 2C), suggesting a pattern of reduced HTT genomic occupancy near SPN identity genes that are transcriptionally downregulated in the striatum across HD model systems.

### Histone methylation and EZH2 occupancy in *Htt^Q111/+^* mice

**Fig. 3.**
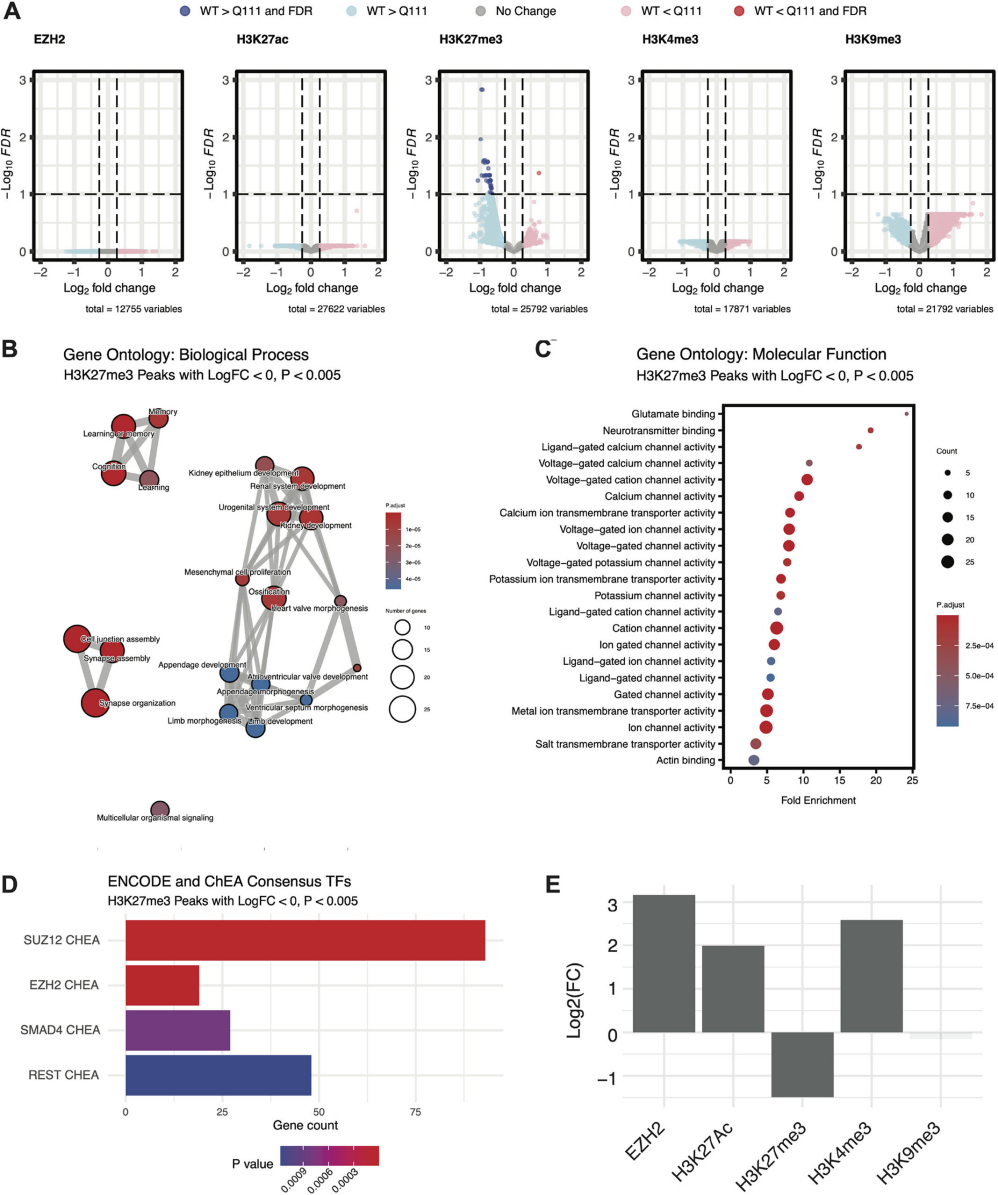
**ChIP-seq analysis reveals early changes in H3K27me3 near developmentally important genes in *Htt^Q111/+^* mice.** (A) Volcano plots summarizing differential histone modifications for H3K27me3, H3K9me3 and H3K4me3, and differential occupancy for EZH2 in striatal tissue from 4-month-old in *Htt^Q111/+^* versus *Htt^+/+^* mice. The *x*-axis indicates the log_2_FC and the *y*-axis indicates the -log_10_ (*P*-value) for the comparison of peak-read depth between genotypes. Regions with greater levels or occupancy in *Htt^+/+^* mice are shown in blue, regions with greater levels or occupancy in *Htt^Q111/+^* mice are shown in pink. Dashed horizontal lines indicate an FDR of 0.1; vertical lines are Log_2_FC=±0.26. (B) Network depiction of Gene Ontology Biological Processes enrichment near differentially methylated regions with reduced H3K27me3 in *Htt^Q111/+^* mice. (C) Gene Ontology Molecular Function enrichment near differentially methylated regions with reduced H3K27me3 in *Htt^Q111/+^* mice. (D) Enrichment of 495 genes in nominal differentially methylated regions with reduced H3K27me3 in *Htt^Q111/+^* mice (unadjusted *P*<0.005) among consensus transcription factor (TF)/target gene databases, i.e. ENCODE and ChEA Consensus TFs from the Enrichr package. Robustly enriched transcription factor lists include: SUZ12 CHEA (93/1684; *P_adj_*=8.3e13; EZH2 CHEA (19/237; *p_adj_*=2.0e04); SMAD4 CHEA (27/584; *P_adj_*=2.6E02); REST CHEA (48/1280; *P_adj_*=2.8e02). (E) Enrichment of EZH2 and indicated chromatin mark ChIP-seq peaks (MACS FDR<0.05) in HTT ChIP-seq peak regions. The *y*-axis indicates the log-transformed fold change (enrichment or depletion) in the number of overlapping base pairs compared to the average of 100,000 re-sampling permutations of genomic coordinates. Plotting density indicates the *P*-value, derived from these same permutations.

**Fig. 4.**
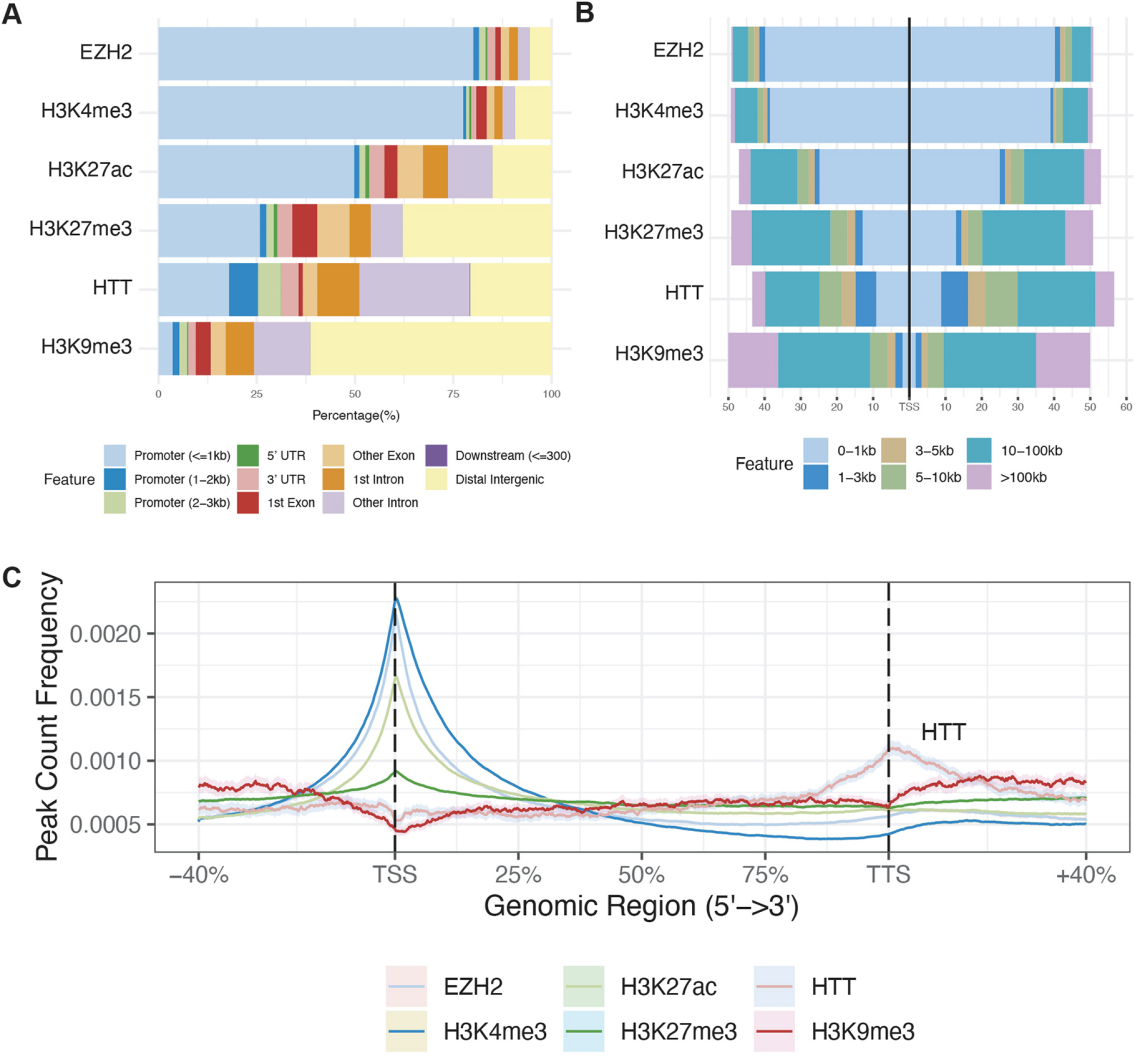
**HTT peaks occupy specific chromatin functional regions.** (A) Summary of genomic regions occupied by HTT and other histone mark and/or EHZ2 occupancy peak sets generated in this study. The percentage of peaks for each dataset is indicated at each vertical line, with boxes indicating the percentage of mark found in each of the categories indicated. (B) Localization of peaks for the indicated chromatin-associated protein at binned distanced from an averaged transcriptional start site (TSS). (C) Peak count frequencies of genic ChIP-seq peaks across the normalized transcript length of all mouse transcripts. The average peak count is indicated, from 5′ (left) to 3′ (right). Genomic regions are indicated as percentages, with 100% spanning from the TSS to the transcriptional termination site (TTS). Shading indicates bootstrapped 95% confidence intervals calculated with resampling (ChIPseeker package).

To compare HTT occupancy and chromatin states of interest, we generated ChIP-seq Histone H3 modifications associated with specific chromatin states: trimethylation of lysine 27 (H3K27me3), trimethylation of lysine 9 (H3K9me3), trimethylation of lysine 4 (H3K4me3) and acetylation of lysine 27 (H3K27ac). These marks are associated with active promoters (H3K4me3), active promoters and enhancers (H3K27ac), facultatively repressed promoters and enhancers (H3K27me3), and constitutively repressed regions (H3K9me3) (Zhou et al., 2011). These experiments used striatal tissue from mice of the same age as the *Htt^Q111/Q111^* animals used in our HTT ChIP-seq experiments described above (*n*=3/genotype) but utilized heterozygous *Htt^Q111/+^* mice to enable matched comparisons to published gene expression datasets from heterozygous *Htt^Q111/+^* mice (Ament et al., 2017; Langfelder et al., 2016; Bragg et al., 2017). After library construction, sequencing, input normalization and peak calling, we identified 17672, 25315, 22304 and 27337 reproducible peaks for H3K4me3, H3K27me3, H3K9me3 and H3K27ac, respectively. As expected, H3K4me3 was localized primarily at promoters, H3K27ac and H3K27me3 in enhancers and promoters, and H3K9me3 in more distal regions (Fig. 4A). Using these data, we examined differential methylation, acetylation or occupancy of each dataset in *Htt^Q111/+^* vs *Htt^+/+^* samples. At FDR<0.1, there were no robust peak differences for H3K9me3, H3K4me3 or H3K27ac, suggesting that any genotypic differences in these marks are subtle. However, we identified 42 H3K27me3 differentially methylated regions (DMRs) at FDR<0.1 (Table S5; Fig. 3A). Notably, 41 of 42 H3K27me3 DMRs (98%) had reduced levels of methylation in *Htt^Q111/+^* vs *Htt^+/+^*mice. At a more lenient *P*-value (*P*<0.05), there were 2018 H3K27me3 DMRs, of which 1926 (96%) showed reduced methylation in *Htt^Q111/+^* samples. These results suggest that HD mutations lead to changes in H3K27 trimethylation in the striatum of 4-month-old *Htt^Q111/+^* mice, including a previously undescribed reduction in H3K27me3 levels in many genomic regions.

The reduction of H3K27me3 in *Htt^Q111/+^* vs *Htt^+/+^* striata suggested a possible partial change of PRC2 function, the enzyme complex responsible for ‘writing’ the H3K27me3 mark (Margueron and Reinberg, 2011). We investigated the genes proximal to H3K27me3 DMRs (*P*-value<0.05) to gain insight into their potential biological consequences. Functional annotation of H3K27me3 DMRs with Genomic Regions Enrichment of Annotations Tool (GREAT) (McLean et al., 2010) and Genekitr (Liu and Li, 2023) (Fig. 3B; Table S6) revealed that negative DMRs in *Htt^Q111/+^* striata were enriched near genes that impinge on development and morphogenesis (e.g. GO:0001822, Kidney Development, *P_adj_*=4.4e-06), genes involved in synapse formation and stability (e.g. GO:0007416, Synapse Assembly, *P_adj_*=1.4e-10), and genes related to cognitive processes (e.g. GO:0007611, Learning or Memory, *P_adj_*=5.3e-07). Genes associated with decreased H3K27me3 levels were enriched for molecular functions at the synapse (Fig. 3C), including glutamate binding (GO:0016595, *P_adj_*=4.8e-04) and calcium channel activity (GO:0005262, *P_adj_*=9.7e-08). Assessment of negative H3K27me3 DMRs for transcription factor-associated chromosomal localization by using Enrichr indicated enrichment at known PRC2 target genes − i.e. those associated with PRC2 subunits EZH2 and SUZ12 (Fig. 3D) − many of which are involved in cell-fate determination (Pereira et al., 2010). Notable examples of developmentally important genes near negative H3K27me3 DMRs include those of transcription factors *Sox11* and *Foxp2.* Therefore, mHTT expression in the striatum is associated with reduced H3K27me3 levels in the vicinity of synaptic, as well as developmentally important genes, including transcription factors that are targets of the PRC2 complex. To further explore this, we performed ChIP-seq of EZH2, the enzymatic component of PRC2 that is responsible for methylation of H3K27, in the striatum of 4-month-old *Htt^Q111/+^* and wild-type mice (*n*=3/genotype), identifying 12,388 reproducible peaks. Interestingly, this analysis revealed no differentially occupied sites (Fig. 3A), suggesting that − at this age − HD does not alter the localization of the EZH2-containing PRC2 complex itself.

We next tested the hypothesis that HTT ChIP-seq peaks colocalize with histone modifications and EZH2 (Fig. 3E;  Table S7). We found a striking overlap between our HTT peaks and EZH2 (log_2_FC=3.2; *P_adj_*=3.2e-05), H3K27ac (log_2_FC=2.0; *P_adj_*=3.2e-05), and H3K4me3 (log_2_FC=2.6; *P_adj_*=3.2e-05). We did not observe global overlap between constitutive heterochromatin marked by histone H3 lysine 9 trimethylation (H3K9me3; log_2_FC=−0.2; *P_adj_*=0.41) but observed a depletion of HTT peaks in H3K27me3 peak regions (log_2_FC=−1.5; *P_adj_*=3.2e-05). This apparent discrepancy between HTT peaks being enriched in EZH2 regions but depleted in H3K27me3 peak regions, might indicate differences in the association of HTT with sites of active EZH2-containing PRC2 complexes versus stably methylated H3K27.

### HTT binds specific chromatin features

To explore a role for HTT in gene regulation, we compared the locations of all 9624 HTT peaks we had observed to known genomic features by using the ChIPseeker Bioconductor package (Yu et al., 2015). Occupied genomic regions of HTT differed substantially compared with the other investigated marks (i.e. H3K4me3, H3K27me3, H3k27ac, H3K9me3) and EZH2, with a notable enrichment within introns and coverage over coding regions of target genes (Figs 4A, 1C,D). Considering the abundance of all peaks relative to the TSS, enrichment of HTT peaks was substantially less near the TSS compared to marks canonically associated with active transcription and open chromatin (i.e. H3K4me3, H3K27ac) and those of EZH2, which also occupied regions upstream of the TSS of target genes, and was less enriched in distal intergenic regions than H3K9me3 (Fig. 4B). HTT peaks had a distinct profile relative to the TSS compared to each of the other mark examined (Fig. 4B) and, of the marks surveyed here, were the only ones showing increased signals near the transcription termination site (TTS; Fig. 4C).

### HTT occupancy predicts gene expression in HD

**Fig. 5.**
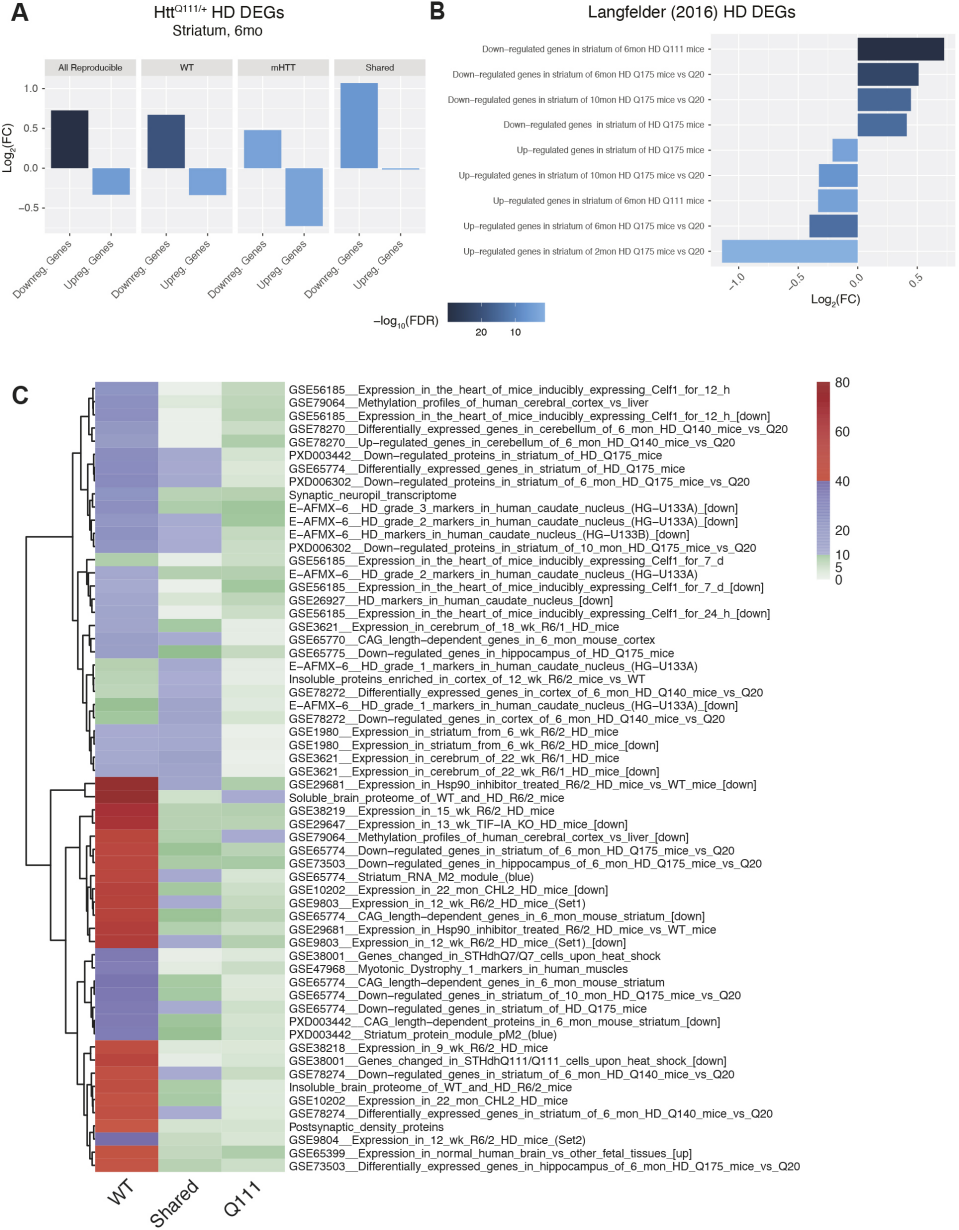
**Genotype-specific HTT occupancy in genomic regions surrounding HD DEGs and other CAG-sensitive gene sets of interest.** (A,B) Enrichment or depletion of HTT ChIP-seq peaks in regions ±20 kb from the transcription start sites of up- and downregulated differentially expressed genes (DEGs) in the striatum of knock-in mouse models of HD mutations, based on RNA-seq from (Langfelder et al., 2016; Rotem et al., 2015). The *y*-axis indicates fold enrichment of base pair overlap compared to the average from 100,000 re-sampling permutations. (C) Heatmap showing HDSigDB gene sets enriched in the regions ±20 kb of WT-specific, Q111-specific, and Shared HTT ChIP-seq peaks. The plotting color indicates the −log_10_(*P*-value) for the strength of the enrichment, based on 100,000 re-sampling permutations. Rows are ordered by hierarchical clustering, based on *P*-values.

The selective enrichment of HTT binding within regions of chromatin bearing marks associated with active transcription (H3K4me3 and H3K27ac; Fig. 4E), and its depletion from a mark associated with repressive transcription (H3K27me3; *ibid*) raised the possibility that HTT directly contributes to HD-related changes in gene expression. We mapped potential HTT target genes whose TSSs were located within ±20 kb of a HTT ChIP peak. Assigning regulatory elements to their target genes is inevitably inexact, but previous work has shown that summing the regulatory elements within 20 kb of a TSS optimizes sensitivity and specificity for detecting gene regulatory interactions (Plaisier et al., 2016). Given the striking occupancy of HTT at specific genes (e.g. Figs 1C,D, 2), we considered whether it might more generally occupy regions near genes that show disease-relevant changes in their expression. We first considered the simplest category of interest: genes that are up- or downregulated in the striatum of HD model mice, with a focus on the *Htt^Q111/+^* striatum of mice aged 6 months, taken from a larger allelic series study (Langfelder et al., 2016). We find enrichment of HTT peaks (irrespective of peak category, or ‘All Reproducible’) near genes that are downregulated in the striatum of 6-month-old *Htt^Q111/+^* mice (Fig. 5A; log_2_FC=0.73, *P_adj_*=1.4e-30) and depletion of HTT peaks near genes that are upregulated (Fig. 5A; log_2_FC=−0.33, *P_adj_*=5.3e-05). Expanding this analysis to differentially expressed genes in other mouse lines and at different timepoints from the allelic series study reinforced our observation that HTT-occupied chromatin peaks are more likely to be associated with downregulated than upregulated genes (Fig. 5B,  Table S8).

We next examined the enrichment of our HTT peaks amongst all manually curated gene sets cataloged in the HDSigDB database (Fig. 5C,  Table S9). As with our DiffBind-nominated peaks with differential binding in *Htt^Q111/Q111^* versus *Htt^+/+^* mice (Fig. 2), this analysis revealed a striking enrichment amongst genes that have been found to be dysregulated by a number of studies and, particularly, in striatal genes that are downregulated in HD (e.g. Fig. 5B). A particularly notable gene set is the ‘Striatum RNA M2 Module (blue)’ from an exhaustive molecular network characterization of an allelic series of HD knock-in mice (Langfelder et al., 2016). This module of genes is notable for being tightly correlated to the length of the (polyQ) CAG tract and the age in the striatum of mHTT-expressing mice, and contains many striatal identity genes. This suggests that our HTT peaks are markedly enriched among striatum-expressed genes, including SPN identity genes that are downregulated in HD. Given the role of PRC2 in regulating cell-specific gene expression and described interactions with HTT (Seong et al., 2010), we specifically focused on PRC2-relevant gene sets in HDSigDB. We found that our shared HTT peaks, i.e. those found in both wild-type and Htt^Q111/Q111^ mice, are strikingly enriched in bivalent genes whose expression is increased in the striatum of 3-month-old (Fig. 5C; Odds ratio=16.7, FDR=7.6e-05) or 6-month-old (Fig. 5C; Odds ratio=6.2, FDR=3.6e-03) mice lacking PRC2 expression in SPNs (*Ezh1^−/−^;Ezh2^−/−^*) (von Schimmelmann et al., 2016). This suggests that our peaks are near genes that are both downregulated in HD as well as upregulated in the context of PRC2 knockout, supporting the hypothesis that HTT binding at a small set of bivalent genes may play an important role in gene dysregulation in HD. Consistent with our own data (Fig. 3D) that reveal a depletion of HTT peaks in H3K27me3 regions, our mutant-specific HTT peaks were depleted near H3K27me3-enriched genes within purified SPNs (Fig. 5C; Odds ratio=16.7, FDR=7.6e-05) (von Schimmelmann et al., 2016).

### Correlations between HTT binding and epigenetic features near differentially expressed genes in HD

**Fig. 6.**
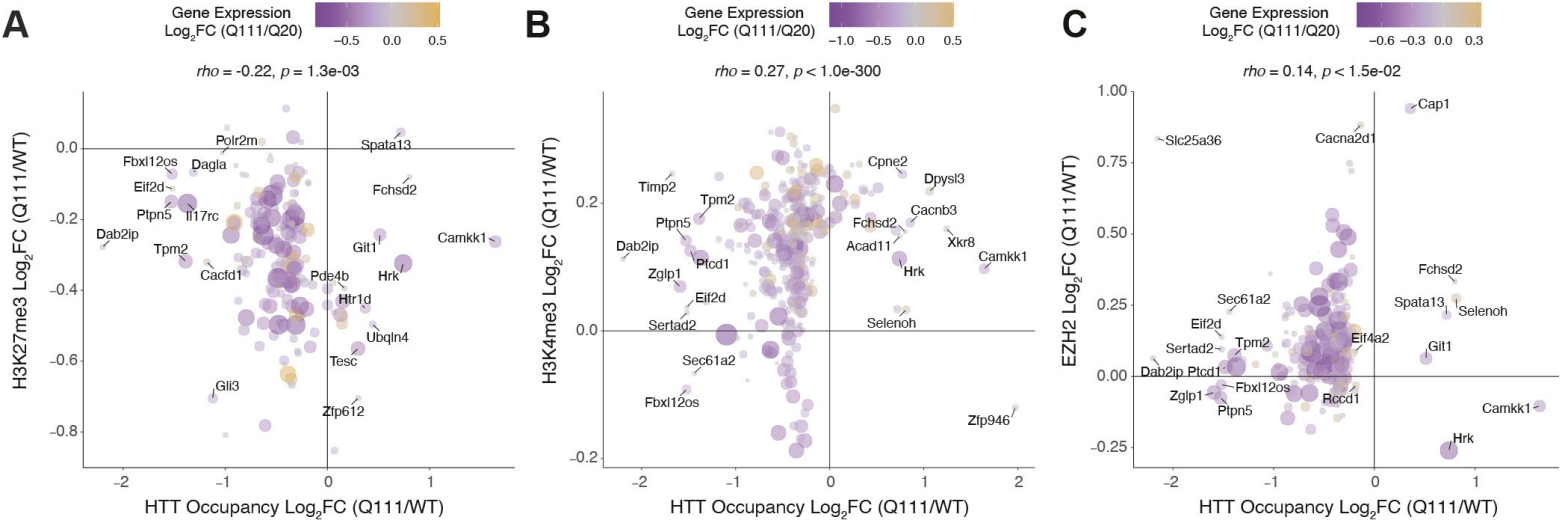
**Increased HTT occupancy is negatively associated with H3K27me3 and positively associated with H3K4me3 near genes that are dysregulated in HD.** (A-C) Each plot indicates the fold change of HTT occupancy (*x*-axis; *Htt^Q111/Q111^* vs *Htt^+/+^*) compared to the fold change in the same regions for H3K27me3 (A), H3K4me3 (B) and EZH2 (C) on the *y*-axis (*Htt^Q111/+^* vs *Htt^+/+^*). Analysis is restricted to 263 HTT peaks located ±20 kb from the TSSs of genes that are significantly up- or downregulated in the striatum of 6-month-old knock-in mouse models of HD mutations (*Htt^Q111/+^* vs *Htt^Q20/+^*). Each circle represents a single HTT ChIP-seq peak. Purple and yellow colored circles indicate the fold change in gene expression in the striatum of heterozygous HD knock-in mice, with upregulated genes in yellow and downregulated genes in purple (see color map). Circle sizes correspond to *P*-values for differential expression.

We hypothesized that the negative correlation between HTT occupancy and HD-related gene expression is mediated by PRC2 and histone modifications. To investigate this, we integrated our data on HTT occupancy, histone modifications and transcription, focusing on the genomic regions proximal to differentially expressed genes (DEGs) in 6-month-old *Htt^Q111/+^* versus *Htt^Q20/+^* mice (Fig. 6A-C; Table S8 (Langfelder et al., 2016). We defined intervals of interest to ±20 kb of the TSS of each DEG in HD and calculated the fold change in *Htt^Q111/Q111^* vs *Htt^+/+^* mice for HTT, H3K27me3, H3K4me3 and EZH2 occupancy within the same interval. Consistent with our hypothesis, the fold changes in H3K27me3 were constitutively lower and correlated negatively with HTT occupancy near DEGs expressed in HD (Fig. 6B) (Spearman's *rho*=−0.22; *P*=1.3e-3). This suggests that *Htt^Q111/+^* mice generally have lower H3K27me3 levels near DEGs in HD, and that increased HTT binding in a region is associated with reduced H3K27me3 in the same interval. By contrast, in those same regions, we observed constitutively higher and positively correlated relationships across genotypes between HTT binding and H3K4me3 (Fig. 6C) (Spearman's *rho*=0.27; *P*<1e-300) and EZH2 (Fig. 6A) (Spearman's *rho*=0.14; *P*=1.5e-2). That is, in *Htt^Q111/+^* mice, levels of EZH2 occupancy and H3K4me3 were generally higher near DEGs in HD, increased HTT binding in that region being associated with more EHZ2 occupancy and higher H3K4me3 levels in that same interval. By contrast, levels of H3K27me3 near DEGs in HD were lower in *Htt*^Q*111/+*^ mice, and greater local HTT binding predicts lower levels of H3K27me3. Importantly, these relationships appeared to be true both for up- and downregulated genes in *Htt^Q111/+^* mice. Thus, concordant changes in PRC2 localization and histone methylation/demethylation may coincide with alterations in HTT occupancy in these regions in both *Htt^Q111/+^* and *Htt^+/+^* mice.

## DISCUSSION

This study was motivated by the question whether transcriptional dysregulation in HD could occur due to interactions between HTT and chromatin. Previously, we have investigated whether specific transcription factors demonstrate differential genomic occupancy and compared this with gene expression changes in *Htt^Q111/+^* mice, finding that the transcription factor SMAD3 demonstrates differential binding between wild-type and mutant mouse brain tissue (Langfelder et al., 2016; Ament et al., 2018). This finding led us to ask what role the HTT protein itself might play with regards to codified chromatin interactions.

ChIP-seq of the HTT protein reveals thousands of robust and reproducible sites of HTT genomic occupancy in the mouse striatum, the brain region most vulnerable to HD pathology and one in which the most pronounced transcriptional dysregulation occurs. Many HTT peaks are enriched across the coding sequence of genes that are of particular interest in HD, with a surprising enrichment near the 3′ end of many transcripts. We further observed, for the first time, a reduction in the levels of H3K27me3 in the striatum of young *Htt^Q111/+^* mice, suggesting these changes represent a very early stage of chromatin alterations in the most vulnerable brain region in HD, i.e. a region that experiences the greatest burden of transcriptional changes in HD. HTT peaks are enriched near genes that are downregulated in HD but are depleted near genes that are upregulated, supporting a link between these HTT−chromatin interactions and bi-directional changes in gene expression in HD. An integrated analysis of HTT binding and histone marks suggests that local HTT binding near DEGs in HD is associated with increased binding of H3K4me3 and EZH2, and reduced levels of H3K27me3.

The HD field has long sought to reconcile the lack of robust phenotypes in mouse models with the length of CAG repeats relevant to adult-onset disease. Recent work studying somatic CAG instability in vulnerable tissue has shown that an expansion of CAG repeats past a critical threshold of ∼150 CAG repeats triggers severe molecular consequences (Handsaker et al., 2025; Wang et al., 2025). While the *Htt^Q111/Q111^* mice used in this study have a baseline CAG repeat lengths in the range of ∼120, they exhibit striatal instability of 1−3.5 additional CAG repeats that are added per month (Lee et al., 2011). Thus, the 4-month-old mHTT-expressing mice used in this study are likely to have a number of SPNs that have reached the critical threshold for enhanced toxicity due to mosaicism. In addition to us finding fewer HTT peaks in homozygous expanded polyQ HTT-expressing mice, we also found that the majority of differential occupancy between wild-type and polyQ expanded HTT is due to loss of HTT (i.e. 244 reduced-occupancy peaks vs four increased-occupancy peaks). Further, genes near the peaks with reduced occupancy were highly enriched for SPN identity markers that have been consistently found to have decreased levels of expression in the striatum of HD model mice. Any roles for HTT interactions with chromatin are not known yet and may mediate facilitatory or inhibitory effects, so our observed expanded polyQ-mediated loss of HTT occupancy is not at odds with the predominantly toxic gain of function etiology of HD.

HTT physically interacts with transcriptional regulatory proteins, such as p53 and CREB-binding protein (CREBBP) (Steffan et al., 2000). In addition, previous ChIP-qPCR experiments with HTT antibodies suggested occupancy in promoter regions of specific genes (Benn et al., 2008). With the improved resolution of genome-wide ChIP-seq, we observed thousands of robust HTT−chromatin interactions in a specific subset of genes. However, these HTT peaks are not primarily localized to the promoters. Instead, distribution of HTT occupancy across genic regions was more reminiscent of marks, such as H3K36me3 (Bannister et al., 2005; Vakoc et al., 2006) and RNAPII Ser2 (Xiao et al., 2007; Barrera et al., 2008), associated with transcriptional elongation and enriched at the 3′ end of the gene. This is a pattern distinct from active marks, such as H3K4me3, which are more strongly associated with TSSs (ENCODE Project Consortium, 2012; Luo et al., 2020). Thus, both gene identity and pattern of HTT binding in those genes argue for a specific selective role for HTT−chromatin interactions. This argument is strengthened by the fact that the specific genes with robust HTT occupancy we profiled within the striatal tissue are enriched for SPN identity genes that we and others have previously found to be strikingly downregulated in HD (Malaiya et al., 2021; Langfelder et al., 2016).

Many genes with HTT peaks identified in our current study are targets of the PRC2 complex, a critical regulator of cell identity via regulation of the H3K27me3 mark near genes important for cell-fate commitment (Margueron and Reinberg, 2011), including in SPNs of the striatum (von Schimmelmann et al., 2016), the most vulnerable brain region to HD pathology (Bates et al., 2015; von Schimmelmann et al., 2016). Direct interactions between HTT and the PRC2 complex have been described, and HTT has been proposed to directly enhance the activity of the enzymatic activity of the PRC2 complex (Seong et al., 2010). The enrichment of HTT within well-annotated PRC2 target genes supports a role for HTT in regulating their expression; however, the occupancy patterns of EZH2 and HTT are quite distinct (Fig. 4A-C). Moreover, while−genome-wide − we observed robust overlap between HTT and EZH2 peaks, we also observed depletion of HTT in H3K27me3 peak regions (Fig. 3D), suggesting that HTT is neither likely to occupy all chromatin-bound PRC2 complexes nor to remain associated with facultative heterochromatin once formed. Given its role in scaffolding functional protein−protein interactions (Seong et al., 2010; Zala et al., 2013), and dynamic shuttling between the nucleus and cytoplasm (Truant et al., 2007), we hypothesize that HTT may play a role in regulating the balance between active and repressed chromatin at select PRC2 target genes.

Several potential mechanisms for the involvement of HTT in transcriptional regulation via the PRC2 complex deserve attention in future studies. A functionally important role has been described for interactions between HTT and the RE1-silencing transcription factor (REST), which plays important roles in repressing neuronal gene expression in non-neuronal cells, (Zuccato et al., 2003). Namely, HTT and REST bind to − and HTT aids in confining REST to − the cytoplasm, thereby preventing aberrant expression of REST target genes in non-neuronal cells (Zuccato et al., 2003). Expression of mHTT results in aberrant accumulation of REST in the nucleus and, consequentially, dysregulation of REST-mediated signaling (Zuccato et al., 2003). With this background, we were intrigued by our results demonstrating that regions with reduced levels of H3K27me3 in the striatum of *Htt^Q111/+^* mice are enriched in both PRC2 and REST target genes (Fig. 3D), especially considering data suggesting that REST may be involved in targeting PRC2 to specific loci (Zuccato et al., 2003; Dietrich et al., 2012). Recently, a new pathway for regulation of PRC2 function via cytoplasmic retention of embryonic ectoderm development (EED), a PRC2 subunit crucial for formation of the complete trimeric structure, has been described (Bodega et al., 2017). In post-mitotic myotubes, a short cytoplasmic form of the PRC2 subunit EZH1 (EZH1β) sequesters EED in the cytoplasm, prevents formation of the PRC2 complex at target genes in the nucleus. Future work focused on understanding whether HTT plays a role in the regulation of PRC2 signaling via cytoplasmic sequestration of PRC2 subunits might be a fruitful area of investigation.

We complemented our HTT ChIP-seq findings with ChIP-seq of histone modifications and EZH2 binding, which revealed reductions in H3K27me3 in the striatum of young *Htt^Q111/+^* mice at levels similar to those seen in *Htt^Q140/+^* mice (Alcalá-Vida et al., 2021) and, particularly, near important striatal cell identity genes (e.g. *Pde10a*). Our differential acetylation data did not recapitulate large-scale genotype-dependent changes in H3K27ac previously seen in the striatum of a HD mouse model (Alcalá-Vida et al., 2021), which could be due to our use of bulk tissue versus fluorescence-activated nuclear sorted neurons. Further, while we did see association of HTT peaks with H3K27ac (Fig. 4E), HTT showed less association than with EZH2 and H3K4me3, suggesting that HTT is not localized to super-enhancers but, instead, to gene bodies (Fig. 3A). The observed reduction in H3K27me3 in *Htt^Q111/+^* mice is consistent with a hypothesis of PRC2 complex dysfunction and/or trafficking disruption occurring at the earliest stages of progression in this model, although other chromatin modifiers could also play roles. The identified lower levels of H3K27me3 seen in heterozygous expanded polyQ HTT-expressing striatum and, in particular, the lack of changes in EZH2 occupancy might implicate H3K27-targeted histone demethylases, such as KDM6A/UTX, in epigenetic changes observed in HD (Song et al., 2018). While correlated, our integrated analysis of HTT, EZH2 binding and histone marks suggests that increased HTT binding near HD DEGs in *Htt^Q111/+^* mice predicts increased binding to H3K4me3 and EZH2, with reduced levels of H3K27me3 at these same loci. We believe that detailed mechanistic experiments focused on elucidating the pathways linking these events could provide important new insights into the role of HTT in transcriptional dysregulation in HD.

This current study has several important limitations. Like all ChIP-seq studies, peak detection is sensitive to background noise, antibody cross-reactivity and other sources of bias. We have tried to control for this by using a relatively large number of technical and biological replicates. Another consideration is the reliance on antibodies, which limits us to establishing chromatin regions associated with specific epitopes of HTT. Future work using phospho-specific antibodies and other dynamic epitopes is likely to nominate additional HTT−chromatin interactions that are of great interest. Functional enrichment analyses of our DiffBind results from wild-type HTT versus mHTT rely on gene assignment that may be biased towards longer genes, propagating these biases through the enrichment tools. This is likely to be less of an issue for HTT since many of its peaks are directly over gene bodies, but highlights that predicting the potential regulatory functions of distal peaks is difficult. Finally, our ChIP-seq experiments relied on bulk striatal tissue, whereas single-nucleus RNA sequencing has indicated bi-directional effects of HD mutations across cell types, especially for PRC2 targets (Malaiya et al., 2021). Emerging techniques enabling ChIP-seq of single cells and sorted cell populations (Malaiya et al., 2021; Rotem et al., 2015) should enable additional refinements of the findings presented here.

While ChIP-on-chip has been performed to identify HTT-binding sites at promoter sequences (Benn et al., 2008), our work provides the first genome-wide map of HTT−chromatin interactions and identified key changes in these interactions due to HD mutations. It suggests that aberrant de-repression of CAG-sensitive genes in HD samples – including cell identity genes – may be due to molecular interactions of HTT and chromatin. We found that altered HTT−chromatin occupancy is accompanied by novel histone modification changes – notably, reductions in H3K27me3 levels – in the striatum of *Htt^Q111/+^* mice, which are associated with genotype-selective HTT occupancy in the same regions. In fact, the strength of HTT occupancy predicts increased H3K4me3 and EZH2 occupancy, and reductions in H3K27me3 levels, consistent with a model in which HTT plays a functional role in regulating PRC2 activity, perhaps by helping recruit PRC2 to specific loci.

## MATERIALS AND METHODS

### Mouse breeding, genotyping and microdissection

The B6.*Htt^Q111^* mice (strain 003456; JAX) used for the ChIP-seq study have a targeted mutation replacing mouse *Htt* exon 1 with the corresponding part of human *HTT* exon 1, including an expanded CAG tract. The targeted *Htt* allele was placed from the CD-1 background onto the C57BL/6J genetic background by selective backcrossing for more than ten generations to the C57BL/6J strain at the Jackson Laboratory (Bar Harbor, ME, USA). Cohorts of homozygote, heterozygote and wild-type littermate mice were generated by crossing B6.*Htt^Q111/+^* and B6.*Htt^+/+^* mice. Male mice were sacrificed at 4 months of age by using a sodium pentobarbital-based euthanasia solution (Fatal Plus, Henry Schein). Brain tissues were snap frozen in liquid nitrogen and stored at −80°C until ChIP was performed. Experiments were performed following the NIH animal care guidelines and approved by the Institutional Animal Care and Use Committee of Western Washington University under protocols 14-005 and 16-011.

### HTT knockdown in cortex

Male mice aged 4 months underwent unilateral intracerebroventricular injection of 500 µg pan-huntingtin antisense oligonucleotide (Ionis, #444652) while naive mice received no treatment. Tissue was collected as above at four weeks post injection; a time shown to have maximal HTT reduction in BACHD mice (Kordasiewicz et al., 2012). Cortical tissue contralateral to the injection was used to assess HTT knockdown by western blot as described (Coffey et al., 2017), ipsilateral tissue was used for ChIP as performed below.

### HTT ChIP-seq

We prepared replicate (*n*=3) ChIP samples using an anti-huntingtin antibody from 4-month-old male *Htt^Q111/Q111^* and age-matched *Htt^+/+^* mice. For each ChIP preparation, chromatin DNA was prepared using the combined striatal tissue from both hemispheres of three mice. Preliminary experiments suggested that this was the minimal amount of material required to provide enough material for multiple immunoprecipitations (IPs). Striata were transferred to a glass Dounce homogenizer on ice and homogenized in cold phosphate-buffered saline with protease inhibitors. For antisense oligonucleotide (ASO) -treated cortices, two cortices were pooled for each replicate (*n*=1). High-resolution crosslinked ChIP-seq (X-ChIP-seq) was performed as previously described (Skene and Henikoff, 2015), with slight modifications (Ament et al., 2018). IPs were performed using 15 µl of Abcam anti-huntingtin EPR5526 (#ab109115). ChIP-seq library preparation and sequencing reactions were conducted at GENEWIZ, Inc. (South Plainfield, NJ, USA). Sequencing was performed on an Illumina HiSeq 4000 using 2×150 Paired End (PE) configuration. Sequencing reads were aligned to the mouse genome (mm10) using HISAT2. Peak-calling was then performed with MACS v2.2, scaling to the size of the input control library. The final set of reproducible peaks was obtained by using the ‘dba’ function in the DiffBind R package (Ross-Innes et al., 2012), retaining peaks that were reproducible at FDR<0.01 in at least two samples and excluding artifactual blacklist regions from ENCODE (ENCODE Project Consortium, 2012). Sequence reads have been deposited in Gene Expression Omnibus, accession GSE150750.

### Histone mark and EZH2 ChIP-seq

Fresh frozen striatal tissue from five male 4-month-old *Htt^Q111/+^* and age-matched *Htt^+/+^* mice were pooled per replicate for *n*=3 samples. Further processing was performed at Active Motif (Carlsbad, CA. USA). Tissues were fixed in 1% formaldehyde, lysed and disrupted with a Dounce homogenizer. DNA was sonicated to an average fragment length of 300−500 bp, and 25 µg chromatin plus 200 ng *Drosophila* spike-in chromatin was incubated with 4 µg antibody targeting EZH2, H3K27ac, H3K27me3 or H3K4me3 (Active Motif catalog numbers 39901, 39133, 39155 and 399159, respectively). Antibody against H2Av was also present in the reaction to ensure efficient pull-down of the spike-in chromatin. Complexes were captured using protein A agarose beads (Invitrogen). Illumina sequencing libraries were prepared from ChIP and Input DNA by end-polishing, 3′ adenylation and adaptor ligation. Libraries were quantified and sequenced using a NextSeq 500 (Illumina) (75nt single end reads).

### Analysis of striatal HTT ChIP-seq data

For the primary striatal HTT ChIP-seq dataset, sequencing reads were aligned to the mouse genome (mm10) using bowtie2 (Langmead and Salzberg, 2012). Peak-calling on each sample was performed with MACS v2.1 (Zhang et al., 2008), scaling each library to the size of the input DNA sequence library to improve comparability between samples. We retained peak regions with a MACS *P*-value of *P*<0.001. Filtered peak calls were concatenated across all *Htt^+/+^* and *Htt^Q111/Q111^* samples to produce a combined set of peak calls and remove peaks overlapping artifactual blacklisted regions of the genome (Amemiya et al., 2019).

### Analysis of cortex HTT ChIP-seq data

Sequencing reads were aligned to the mouse genome (mm10) using HISAT2. Aligned reads from the four ChIP libraries were down-sampled to the size of the smallest sequencing library using Samtools-view -s (Li et al., 2009). Aligned reads from the input genomic DNA of all four samples were merged into a single control BAM file. Peak-calling was then performed with MACS2.2, scaling to the size of the input control library. The final set of reproducible peaks was obtained using the dba function in the DiffBind R package, retaining peaks that were reproducible at FDR<0.01 in at least two samples and excluding artifactual blacklist regions from ENCODE (ENCODE Project Consortium, 2012). We then used dba.count to count the reads in each peak region from each ChIP sample and from the control sample. Next, a generalized linear model was fit, using the dba.analyze DESeq2 wrapper, and we performed log-ratio tests to estimate effects of ASO treatment on HTT occupancy in each peak region. Genotype was treated as a blocking factor. Control read counts were subtracted for each site in each sample before fitting the model. This experiment was underpowered to detect statistically significant changes in occupancy at individual peak regions. Therefore, our primary test was for global depletion of HTT occupancy in ASO-treated mice across peak regions. For this purpose, we computed one-sided binomial tests with binom.test, comparing the number of peaks with increased vs decreased occupancy.

### Analysis of histone mark and EZH2 ChIP-seq

Reads were aligned consecutively to the mouse (mm10) and *Drosophila* (dm3) genomes using the Burrows-Wheeler Aligner (BWA) algorithm (default settings). The number of mouse alignments used in the analysis was scaled to the number of *Drosophila* spike-in alignments (Egan et al., 2016). Peak-calling was performed with MACS v2.1. We selected peaks that were reproducibly identified in at least two samples of the same genotype. For analyses of differential occupancy, reads mapped to each peak region were normalized to total library size. Generalized linear models and log-ratio tests were fit with edgeR (Robinson et al., 2010) to identify differentially methylated regions.

### Enrichment of peaks in genomic regions and gene sets

Over-representation of HTT peaks in chromatin states, genomic regions marked by histone modifications, and gene sets were calculated using the Genomic Association Tester (GAT) (Heger et al., 2013). GAT calculates the number of base pairs overlapping between two genomic annotations and estimates its fold enrichment and significance based on re-sampling permutations within the mappable genome. Results described in this manuscript are based on 100,000 re-sampling permutations. Accession numbers for comparison datasets are shown in the Key Resources Table. For ChromHMM and ChIP-seq comparison datasets, we downloaded published tables of peak regions from the ENCODE portal or from the Gene Expression Omnibus. Similarly, for analyses of gene sets, we downloaded gene lists from HD Molecular Signatures (HDSigDB) and defined regions of interest as ±20 kb of the canonical TSSs of the genes in each set.

## Supplementary Material

10.1242/dmm.052282_sup1 Supplementary information

Table S1. Significant HTT peak calling results, including proximal gene names, including genotype category labels. The “MACS Significant HTT Peaks” tab includes the MACS peak calling results - annotated with the most proximal gene and the distance to it. The “Peak.Label” column indicates whether each peak is in the category of “mHTT-specific”, “WT-specific”, or “WT-mHTTshared” - see text for details on these categories.

Table S2. HTT Peaks Per Gene. A per-gene summary of HTT peaks across the categories of “mHTT-specific”, “WT-specific”, or “WT-mHTT-shared.”

Table S3. HTT DiffBind. Differential HTT occupancy in *Htt^Q111/Q111^ vs. Htt^+/+^*. Used to generate volcano plot Fig. 2A.

Table S4. HTT DiffBind Enrichr Results. Geneset enrichment results for differential HTT peak occupancy in Figs. 2B, 2C.

Table S5. DiffBind Summary ActiveMotif. Differential occupancy of EZH2, H3K27ac, H3K27me3, H3K4me3, and H3K9me3, peak regions. These results were used to generate Fig. 3A.

Table S6. H3K27me3 Enrichment. Enrichment of H3K27me3 peaks with lower occupancy in *Htt^Q111/+^ vs. Htt^+/+^* striatum (p < 0.005) assessed for enrichment using Enrichr. Tabs correspond to terms for Gene Ontology Biological Process (GOBP), Gene Ontology Molecular Function (GOMF), and ENCODE and ChEA Consensus Transcription Factors (ENCODE-CHEA_tfs). Used to generate Figs. 3B-D.

Table S7. HTT Enrichment ActiveMotif Peaks. Summary of GAT results testing the enrichment of HTT ChIP-seq peaksets amongst EZH2, H3K27ac, H3K27me3, H3K4me3, and H3K9me3, peak regions. These results were used to generate Fig. 3E.

Table S8. HTT Peaks, ActiveMotif and RNASeq Integration. Includes integrated ChIP-seq and RNA-seq data for all intervals containing robust HTT peaks. For each HTT peak (“PeakRegion.ID”), available changes in RNA expression of included genes (“Langfelder et. al. RNA-Seq”) and our other ChIP-seq data (“Re-analysis of ActiveMotif ChIP-Seq”) are shown. These results were used to generate Fig. 5A-B and 6A-C.

Table S9. HTT HDSigDB Overlap. Includes enrichments for HTT Peak sets and the genes included in each HDSigDB gene set. HDSigDB gene set meta information is included on the tab “HDSigDB.Genesets”. These results were used to generate Fig. 5C.
