# Supplementary material for: Altered huntingtin−chromatin interactions predict transcriptional and epigenetic changes in Huntington's disease
Source: Dis Model Mech. 2025 May 27;18(5):dmm052282. doi: 10.1242/dmm.052282 (PMC12147460; doi:10.1242/dmm.052282)
Supplement: Supplementary information [file dmm-18-052282-s1.pdf]

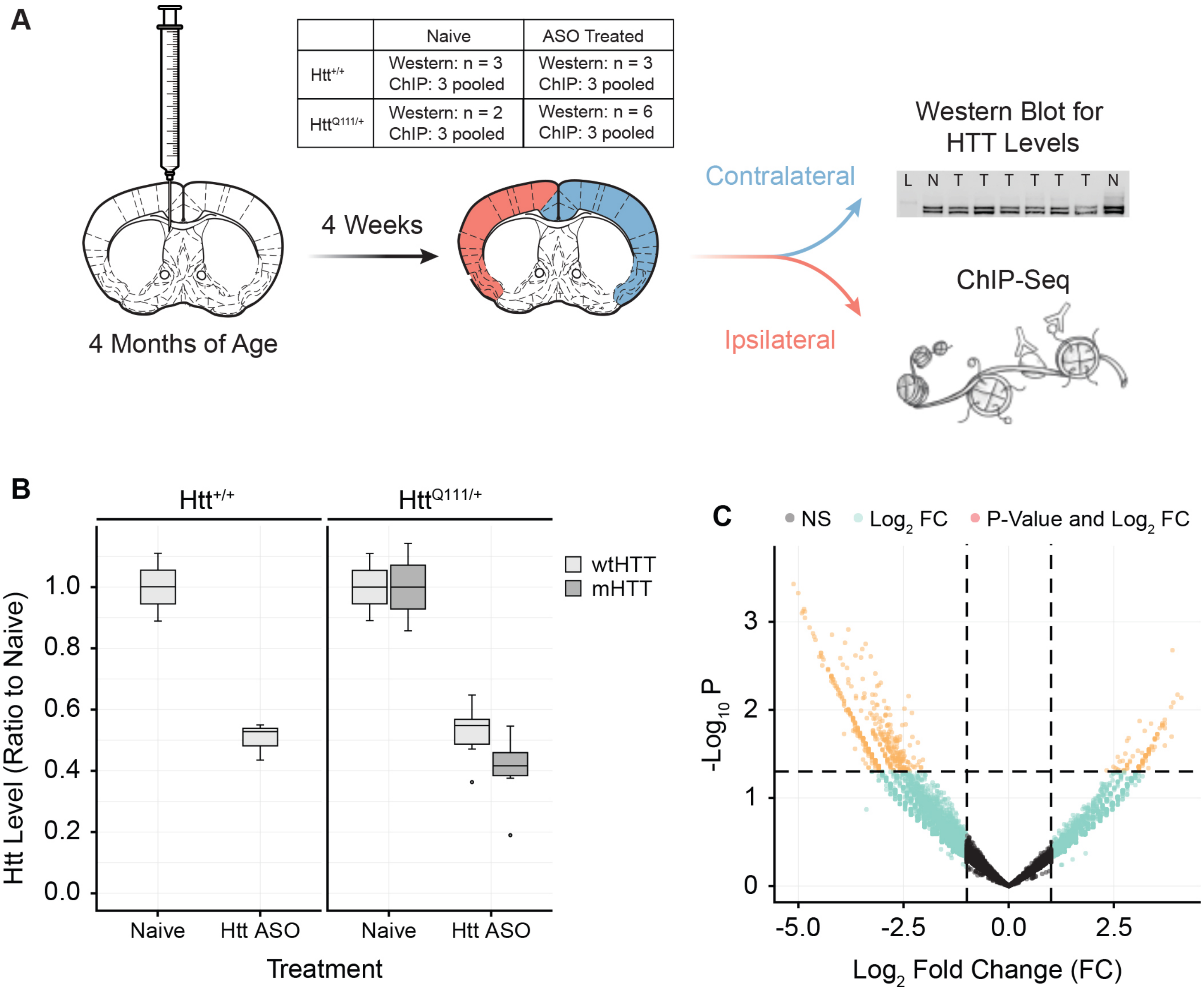

Fig. S1. Treatment with a HTT-lowering ASO reduces HTT ChIP-seq peaks. A) Mice underwent unilateral intracerebroventricular injection with HTT-targeted ASO. Cortex ipsi- and contralateral to the injection were collected at four weeks post-injection. HTT lowering was assessed in the contralateral cortex, while ChIP was performed on the ipsilateral cortex. B) Quantification of western blots probed with anti-HTT EPR5526 show HTT lowering of 53% in the contralateral cortex at four weeks post-ASO injection (ANOVA, Treatment effect p = 4.11e09). C) Volcano plot demonstrating 13,820 reproducible HTT-associated peaks in the cortex, identified in either genotype, 8,308 (60%) showing Log_2(fold change) < 0 with ASO treatment. Likelihood that more than 50% had negative fold changes, p = 4.2e126 (binomial test). Of differentially occupied peaks ($p < 0.05$): 502 out of 622 had negative fold changes (80%) with ASO treatment. Despite the small sample size and partial HTT knockdown, we detected a global depletion of HTT occupancy in the cortex of mice treated with HTT-lowering ASOs.

Disease Models & Mechanisms • Supplementary information

Table S1. Significant HTT peak calling results, including proximal gene names, including genotype category labels. The “MACS Significant HTT Peaks” tab includes the MACS peak calling results - annotated with the most proximal gene and the distance to it. The “Peak.Label” column indicates whether each peak is in the category of “mHTT-specific”, “WT-specific”, or “WT-mHTT-shared” – see text for details on these categories.

Available for download at
https://journals.biologists.com/dmm/article-lookup/doi/10.1242/dmm.052282#supplementary-data

Table S2. HTT Peaks Per Gene. A per-gene summary of HTT peaks across the categories of “mHTT-specific”, “WT-specific”, or “WT-mHTT-shared.”

Available for download at
https://journals.biologists.com/dmm/article-lookup/doi/10.1242/dmm.052282#supplementary-data

Table S3. HTT DiffBind. Differential HTT occupancy in $Htt^{Q111/Q111}$ *vs. Htt*$^{+/+}$. Used to generate volcano plot Fig. 2A.

Available for download at
https://journals.biologists.com/dmm/article-lookup/doi/10.1242/dmm.052282#supplementary-data

Table S4. HTT DiffBind Enrichr Results. Geneset enrichment results for differential HTT peak occupancy in Figs. 2B, 2C.

Available for download at
https://journals.biologists.com/dmm/article-lookup/doi/10.1242/dmm.052282#supplementary-data

Disease Models & Mechanisms • Supplementary information

Table S5. DiffBind Summary ActiveMotif. Differential occupancy of EZH2, H3K27ac, H3K27me3, H3K4me3, and H3K9me3, peak regions. These results were used to generate Fig. 3A.

Available for download at
https://journals.biologists.com/dmm/article-lookup/doi/10.1242/dmm.052282#supplementary-data

Table S6. H3K27me3 Enrichment. Enrichment of H3K27me3 peaks with lower occupancy in *Htt*$^{Q111/+}$ *vs. Htt*$^{+/+}$striatum ($p < 0.005$) assessed for enrichment using `Enrichr`. Tabs correspond to terms for Gene Ontology Biological Process (GOBP), Gene Ontology Molecular Function (GOMF), and ENCODE and ChEA Consensus Transcription Factors (ENCODE-CHEA_tfs). Used to generate Figs. 3B-D.

Available for download at
https://journals.biologists.com/dmm/article-lookup/doi/10.1242/dmm.052282#supplementary-data

Table S7. HTT Enrichment ActiveMotif Peaks. Summary of `GAT` results testing the enrichment of HTT ChIP-seq peaksets amongst EZH2, H3K27ac, H3K27me3, H3K4me3, and H3K9me3, peak regions. These results were used to generate Fig. 3E.

Available for download at
https://journals.biologists.com/dmm/article-lookup/doi/10.1242/dmm.052282#supplementary-data

Table S8. HTT Peaks, ActiveMotif and RNASeq Integration. Includes integrated ChIP-seq and RNA-seq data for all intervals containing robust HTT peaks. For each HTT peak (“PeakRegion.ID”), available changes in RNA expression of included genes (“Langfelder et. al. RNA-Seq”) and our other ChIP-seq data (“Re-analysis of ActiveMotif ChIP-Seq”) are shown. These results were used to generate Fig. 5A-B and 6A-C.

Available for download at
https://journals.biologists.com/dmm/article-lookup/doi/10.1242/dmm.052282#supplementary-data

Table S9. HTT HDSigDB Overlap. Includes enrichments for HTT Peak sets and the genes included in each HDSigDB gene set. HDSigDB gene set meta information is included on the tab “HDSigDB.Genesets”. These results were used to generate Fig. 5C.

Disease Models & Mechanisms • Supplementary information
